# 

**Published:** 1910-02-05

**Authors:** 


### Gifts and Bequests.

Mr. James Whitehead has left £200 to the Oldham Infirmary.

The Middlesex Hospital has received £100 from "R. D. W."

Sir John Llewelyn has given £1,000 to Swansea Hospital building fund.

Mr. Reginald Alexander Thomson, of Newcastle and Blackpool, has left £500 to the Oldham Infirmary.

Mr. Henry Thomas Curling, R.A., of Ramsgate, has left £100 to the Ramsgate General Hospital.

The Hospital for Sick Children has received £100 from the executors of the late Lady Campbell Clarke.

Mr. Henry Bayly Garling, F.R.I.B.A., of Folkestone, has left £100 to St. George's Hospital, Hyde Park.

The Hon. William Ellis Gloag, Lord Kincairney, of Edinburgh, and of Kincairney, Perthshire, has left £30 to the Perth Infirmary.

The Cleckheaton Co-operative Society has given £250 to the Bradford New Infirmary scheme, and the Brighouse Co-operative Society £52 10s.

Sir Thomas Freeman Firth, chairman of Messrs. T. F. Firth and Sons, Ltd., blanket and carpet manufacturers, has left £500 to the Dewsbury Infirmary.

### Appointments.

The following re-appointments have been made by the Board of Management of the Manchester Royal Infirmary: F. E. Tylecote, M.D. (Vict.), M.R.C.P., resident medical officer, Manchester Royal Infirmary; D. E. Core, M.B., Ch.B. (Vict.), resident medical officer, Barnes Convalescent Home; G. E. Loveday, M.B., B.C. (Cantab.), M.R.C.S., L.R.C.P., director of the clinical laboratory; A. F. Thompson, M.B., Ch.B. (Vict.), senior anæsthetist; S. R. Wilson, M.B., B.S. (Lond.), M.B., Ch.B. (Vict.), second anæsthetist; R. A. H. Atkinson, M.B., Ch.B. (Vict.), junior anæsthetist; A. F. Barclay, M.A., B.C. (Cantab.), M.R.C.S., L.R.C.P., medical officer for radiography and electricity department; J. P. Buckley, B.A. (Cantab.), M.B., B.S. (Lond.), J. Morley, M.B., Ch.B. (Vict.), medical officers, Central Branch; F. G. Wrigley, M.B., Ch.B. (Vict.), gynæcological house surgeon. E. B. Leech, M.D. (Vict.), M.R.C.P., medical registrar, has been appointed.

### THE QUESTION BOX.

**SPECIAL NOTICE.**—Questions of interest affecting the honorary medical staff and hospital administration in all departments are invited. When any point arises we hope our readers will formulate it in a question and so co-operate to make this column of real value and interest. In this way the experience of the best-managed hospitals should be made helpful to the whole hospital world. We shall welcome the contribution of both questions and answers.

N.B.—The publication of an answer in this column does not necessarily preclude the publication of subsequent answers to the same question, for a question may involve points which require to be threshed out and fully discussed. Answers, if published, will be paid for at scale rates.

### WANTED, A SYSTEM FOR FILING.

Question: What is the best system for filing records, documents, and letters suitable for a large hospital?

### A London View.

By An Old Hospital Secretary.

Unless the hospital is a very large one it is better not to attempt any elaborate system of filing. All office letters should be put away in bundles, arranged alphabetically, once a month, and the letter book will form to these a complete index. An improvement on this is to adopt one of the Shannon files, or any similar method of keeping letters alphabetically arranged, but, as before, trusting to the letter book as an index.

This simplifies the work in the office, and even with the very heaviest correspondence it is rare that a letter is not forthcoming when it is needed.

### A Cardiff View.

By Mr. A. Ernest Wilkes, Assistant Secretary, Cardiff Infirmary.

The best system of filing records, etc., for a large hospital is undoubtedly the vertical system (with folders), filing according to subject, as it is so readily adaptable to any fresh demand that may be made upon its resources by the introduction of further folders for new subjects. To facilitate reference it is necessary that, in addition to the filing cabinet, a means of cross-indexing be provided, and a card index serves admirably for this purpose. The subject matter is written on the index cards, which are filed alphabetically. For example—"By-laws" under "B." This card would have written on it the information that By-laws for Resident Medical Officer are filed under "B" and in folder No. 21, By-laws for Nurses under "B" and in folder No. 22, and so on. The cross index cards are then written; for example, Resident Medical Officer (By-laws), Nurses (By-laws), which are filed under "R" and "N" respectively, and contain the information already given on the "By-laws" card—namely, that the By-laws for these offices are filed under "B" and in folders 21 and 22. So much for the index. As regards the filing cabinet itself, the papers relating to each subject are accommodated in separate folders, on the tabs of which the names of the subjects are inscribed. These folders are placed behind alphabetical guide cards and numbered. Should it be necessary to remove temporarily any paper from the cabinet this can easily be done, as the papers are not fastened in. In this case a note should be made and placed in the folder, giving the date when the documents were taken out and the name of the person to whom they were entrusted.

The advantages of vertical filing are many, as papers may be easily removed or replaced without disarranging other papers in the cabinet. Copies of minutes and resolutions, as well as notes, may be placed in the folders, thereby saving valuable time in future references when it may obviate direct reference to the original resolutions, etc. Many letters received by a hospital are practically valueless after a couple of years; but if it is thought undesirable to have them destroyed, they may be stored in numbered parcels, the distinctive number of each parcel, together with date of removal, being written on the index card and folder, which remains in the cabinet.

This system may be applied to any and every interest affecting the administration of a hospital. For instance, price lists, quotations, references to samples of goods or material, etc., may be referred to without waste of time; samples, if bulky, being numbered and dealt with in the same way as parcels.

### A Birmingham View.

By Mr. C. A. Mason, Secretary, Birmingham and Midland Eye Hospital.

In hospitals where prescription papers are retained, I think the best system is to file them vertically in numerical order, the number being stamped on the left-hand top-corner with an automatic numerator capable of stamping the same number as many times as the particular circumstances of the hospital may require. This can be effected by the registrar on the patient's first attendance, the same number being also recorded on a card, which is handed to the patient for presentation on subsequent visits; and again on the ticket (if it be a ticket hospital), or in a free-case list, thus automatically recording the number of new (ticket and free) cases by means of practically one operation. An alphabetical card-index is essential to a numerical system of filing owing to the fact that patients lose the cards bearing their number.

I also use the card-index system for the collating of medical statistics. A guide card is used for every disease, the diagnosis being taken from the place reserved for it on the prescription paper, and entered, together with the surgeon's name and patient's number, on a 3 by 5 card. Each entry is also given a progressive number, so that at the end of the year the last number on the last card in each diagnosis is the number of cases of that disease treated during the period. By this means, too, a surgeon can obtain the whole of his papers relating to any particular disease, should it be desirable for clinical information

For filing purposes I have found the Library Bureau cabinets work admirably, being well made and capable of easy expansion. Each double section has six drawers which accommodate about 15,000 prescription papers and charts, and eight drawers accommodating about 32,000 3 by 5 index-cards. The one disadvantage of the system is the vast accumulation of papers, many of which have little if any clinical value, which is involved. This, however, could doubtless be dealt with according to the special circumstances of the particular institution. At this hospital a surgeon stamps those papers worthy of permanent retention with the word "Retain," and others are periodically destroyed after the lapse of two years.

Our patients used to take their papers away, but though the retention of them certainly involves more labour, the cost, including the necessary cabinets, is not appreciably increased, whilst the advantages in cleanliness and efficiency are unquestionable.

THE

### GRADUATES' MEDICAL SCHOOLS.

DIARY FOR THE WEEK, FEBRUARY 7 to 12.

**THE BROMPTON HOSPITAL FOR CONSUMPTION AND DISEASES OF THE CHEST, Fulham Road, S.W.**

**At 4 p.m.—Feb. 9,** Dr. Hartley, **Clinical Varieties of Pulmonary Tuberculosis and their Treatment.**

**ST. JOHN'S HOSPITAL FOR DISEASES OF THE SKIN, Leicester Square, W.C.**

**At 6 p.m.—Feb. 10,** Chesterfield Lecture: **Tuberculosis of the Skin.**

**LONDON SCHOOL OF CLINICAL MEDICINE, Seamen's Hospital, Greenwich, S.E.**

**At 4 p.m.—Feb. 7,** Mr. R. Lake, **Indications for Mastoid Operation.**
**2.15 p.m.—Feb. 9,** Dr. F. Taylor, **Diabetes.**

**THE HOSPITAL FOR SICK CHILDREN, Great Ormond Street, W.C.**

**At 4 p.m.—Feb. 10,** Dr. F. Batten, **Selected Medical Cases.**

**NORTH-EAST LONDON POST-GRADUATE COLLEGE, Prince of Wales's General Hospital, Tottenham, N.**

**At 4.30 p.m.—Feb. 8,** Dr. G. N. Meachen, **Selected Skin Cases.**
**Feb. 10,** Mr. E. Gillespie, **Chronic Gonorrhœa and its Sequelæ.**

**MEDICAL GRADUATES' COLLEGE AND POLYCLINIC, 22 Chenies Street, W.C.**

**At 4 p.m.—Feb. 7,** Dr. Graham Little, **Clinique (Skin).**
**5.15 p.m.—Feb. 7,** Mr. Chas. A. Morton (Bristol), **Removal of Stones from the Ureter.**
**4 p.m.—Feb. 8,** Dr. G. A. Sutherland, **Clinique (Medical).**
**5.15 p.m.—Feb. 8,** Dr. George Pernet, **Extra-Genital Chancres.**
**4 p.m. Feb. 9,** Mr. Cecil H. Leaf, **Clinique (Surgical).**
**5.15 p.m.—Feb. 9,** Dr. Charles Mercier, **Cases of Mental Disorder.**
**4 p.m.—Feb. 10,** Sir Jonathan Hutchinson, **Clinique (Surgical).**
**5.15 p.m.—Feb. 10,** Dr. J. H. Sequeira, **Bullous Eruptions.**
**4 p.m.—Feb. 11,** Mr. Harold Barwell, **Clinique (Ear, Nose, and Throat).**

**CENTRAL LONDON THROAT AND EAR HOSPITAL, Gray's Inn Road, W.C.**

**At 3.45 p.m.—Feb. 8,** Mr. Gay French, **Tracheoscopy, &c.**
**3.45 p.m.—Feb. 11,** Dr. Wyatt Wingrave, **Clinical Pathology.**

**THE POST-GRADUATE COLLEGE, West London Hospital, Hammersmith, W.**

**At 10 a.m.—Feb. 7 and Feb. 10, Demonstration by Surgical Registrar.**
**10 a.m.—Feb. 11, Demonstration by Medical Registrar.**
**12 noon.—Feb. 10,** Dr. Bernstein, **Pathological Demonstration.**
**12.15 p.m.—Feb. 9,** Dr. Grainger Stewart, **Practical Medicine.**
**5 p.m.—Feb. 7,** Mr. Baldwin, **Practical Surgery.**
**5 p.m.—Feb. 8,** Dr. Seymour Taylor, **The Serious Valve Diseases of the Heart.**
**5 p.m.—Feb. 9,** Mr. Lloyd Williams, **Local Anæsthetics.**
**5 p.m.—Feb. 10,** Mr. Edwards, **Urinary Surgery.**
**5 p.m.—Feb. 11,** Dr. Cole, **Insanity at the Decline of Life.**

**ROYAL SOCIETY OF MEDICINE, 20 Hanover Square, W.**

**At 5.30 p.m.—Feb. 8, Surgical Section:**

*Papers.*—Mr. Ernest W. Hey Groves: "Radical operation for cancer of the pylorus" (illustrated by the epidiascope). Mr. Somerville Hastings: "Rupture of the tunica vaginalis in hydroceles."

**At 7.45 p.m.—Feb. 10, Obstetrical and Gynæcological Section:**

*Specimens.*—Dr. Russell Andrews: Fibroma of the vagina. Dr. Jervois Aarons: Ectopic gestation. Dr. Hastings Tweedy: Ovarian pregnancy. Mr. Hey Groves: Tubal mole. Dr. A. H. Lewers: Uterus ruptured transversely at fundus, during labour.

*Short Communication.*—Dr. William Alexander: Post-peritoneal hæmatocele complicating a large uterine fibroid.

*Paper.*—Dr. Hastings Tweedy: "Modern methods of delivery in contracted pelves."

**At 8 p.m.—Feb. 11, Clinical Section:**

*Clinical Cases.*—Dr. Essex Wynter: Angioneurosis. Dr. Galloway: Angioneurosis. Mr. Morley Fletcher: Two cases of rheumatic nodules with gonococci. Dr. Langmead: Hereditary multiple telangiectases. Mr. Carless: (1) Sarcoma of the innominate bone under treatment by Coley's fluid; (2) Removal of both jaws for epithelioma, extension to frontal bone. Dr. St. Clair Thomson: Four cases of epithelioma of the larynx after operation. And other cases.

*Paper.*—Dr. Essex Wynter: "Aneurysm of the descending thoracic aorta."

### APPOINTMENTS VACANT.

Liverpool Hahnemann Hospital and Dispensary.—Non-resident stipendiary medical officer.

The *Strand Magazine* recently offered a prize of £50 for the best medical story, either fact or fiction, which was to be written by a qualified medical man, and which had to be sufficiently interesting and well-written to justify its publication on its own merits. In response to this offer large numbers of stories were sent in, and the February issue of the *Strand* contains the story which has been adjudged to be the best. This is a short story by Mr. J. Bart Rous, M.R.C.S., L.R.C.P., entitled "Under the Microscope," which gives an account of an impressionable young man who was put to great torture of mind by the mistake of a pathologist who had mixed his slides. The impressionable young man has a simple lingual ulcer, of which the surgeon agrees to excise a bit for microscopic examination. The slide is returned with a note from the eminent pathologist that the specimen is carcinomatous. When the tragic interest is at its height in rushes the eminent pathologist and declares that he has mixed the slides. The story is crude and unsatisfactory in the extreme, and displays no great professional intelligence, but it has some interesting points. In time and with practice Mr. Rous will doubtless evolve very much better literary attempts.

THE HOSPITAL. February 5, 1910.

*Name* ........................................

*Address* ........................................

........................................

**This Coupon must accompany manuscript or contributions intended for The Hospital.**

